# Fine-Scale Source Apportionment Including Diesel-Related Elemental and Organic Constituents of PM_2.5_ across Downtown Pittsburgh

**DOI:** 10.3390/ijerph15102177

**Published:** 2018-10-05

**Authors:** Brett J. Tunno, Sheila Tripathy, Ellen Kinnee, Drew R. Michanowicz, Jessie LC Shmool, Leah Cambal, Lauren Chubb, Courtney Roper, Jane E. Clougherty

**Affiliations:** 1 University of Pittsburgh Graduate School of Public Health, Department of Environmental and Occupational Health, Pittsburgh, PA 15219, USA; sheila.tripathy@gmail.com (S.T.); ejk40@pitt.edu (E.K.); drewmichanowicz@gmail.com (D.R.M.); jlcshmool@gmail.com (J.L.C.S.); ltipton86@gmail.com (L.C.); lgc4@pitt.edu (L.C.); clr56@pitt.edu (C.R.); jec373@drexel.edu (J.E.C.); 2 Drexel University Dornsife School of Public Health, Department of Environmental and Occupational Health, Philadelphia, PA 15219, USA

**Keywords:** diesel, elemental constituents, factor analysis, fine particulate matter, organic compounds, source apportionment

## Abstract

Health effects of fine particulate matter (PM_2.5_) may vary by composition, and the characterization of constituents may help to identify key PM_2.5_ sources, such as diesel, distributed across an urban area. The composition of diesel particulate matter (DPM) is complicated, and elemental and organic carbon are often used as surrogates. Examining multiple elemental and organic constituents across urban sites, however, may better capture variation in diesel-related impacts, and help to more clearly separate diesel from other sources. We designed a “super-saturation” monitoring campaign of 36 sites to capture spatial variance in PM_2.5_ and elemental and organic constituents across the downtown Pittsburgh core (~2.8 km^2^). Elemental composition was assessed via inductively-coupled plasma mass spectrometry (ICP-MS), organic and elemental carbon via thermal-optical reflectance, and organic compounds via thermal desorption gas-chromatography mass-spectrometry (TD-GCMS). Factor analysis was performed including all constituents—both stratified by, and merged across, seasons. Spatial patterning in the resultant factors was examined using land use regression (LUR) modelling to corroborate factor interpretations. We identified diesel-related factors in both seasons; for winter, we identified a five-factor solution, describing a bus and truck-related factor [black carbon (BC), fluoranthene, nitrogen dioxide (NO_2_), pyrene, total carbon] and a fuel oil combustion factor (nickel, vanadium). For summer, we identified a nine-factor solution, which included a bus-related factor (benzo[ghi]fluoranthene, chromium, chrysene, fluoranthene, manganese, pyrene, total carbon, total elemental carbon, zinc) and a truck-related factor (benz[a]anthracene, BC, hopanes, NO_2_, total PAHs, total steranes). Geographic information system (GIS)-based emissions source covariates identified via LUR modelling roughly corroborated factor interpretations.

## 1. Introduction

Diesel particulate matter (DPM) has been classified as a probable human carcinogen by the United States Environmental Protection Agency (U.S. EPA), [1,2,3,4] and has been associated with lung injury and respiratory health [4], cardiovascular illness, birth outcomes [5], cognitive effects [6,7], blood-brain barrier integrity [8], endocrine disruption [9], and other health outcomes, in epidemiologic and controlled-dose studies in both humans [10,11,12] and animals [13,14,15,16]. In addition, recent evidence indicates that diesel emissions may be a major contributing factor to climate change [17]. By more clearly identifying the air pollution impact of diesel sources in urban areas, researchers can more accurately evaluate diesel-related health effects, and interventions may be more effectively targeted towards reducing population exposures and improving urban health.

The contribution of DPM to local fine particulate matter (PM_2.5_) and health can be challenging to isolate using standard sampling instrumentation or analytic techniques [18]. This challenge is due, in part, to the complexity of PM_2.5_—a composite of elemental and organic constituents which varies in composition both between and within urban areas. There is substantial spatial variation in diesel source densities, and variation by time of day, owing both to differences in source density (e.g., rush hours) and meteorology as relates to dispersion, mixing height, and transformation to secondary species. As regulatory and technological changes have led to changes in diesel emissions over recent years, isolating the diesel-attributable portion of urban air pollution is profoundly complex. As a result, there remain substantial uncertainties in evaluating the emissions-reduction potential of policies aimed at reducing diesel-pollution exposures in urban areas, hindering policy development and related cost-benefit analyses.

Lacking strong indicators of ambient DPM, organic carbon (OC) and elemental carbon (EC) are often used as imperfect exposure surrogates, because DPM is approximately 80% carbon [19,20,21]. Factor analytic source apportionment analysis, however, which identifies highly-correlated suites of constituents and pollutants [22,23] can enable identification and analysis of source-specific PM_2.5_ with adverse health outcomes [24].

Land use regression (LUR) modeling—which quantifies spatial associations between geographic information system (GIS)-based source density indicators and measured concentrations—can identify key sources spatially associated with specific constituents or constituent suites (e.g., factor scores) [22]. In this way, LUR models can be used in combination with traditional factor analytic source apportionment methods, corroborating the interpretation of derived factors [25].

We previously reported that bus density explained substantial spatial variation in PM_2.5_, BC, EC, OC, and various other organic and elemental constituents [26] across the downtown Pittsburgh core. Here, we merge factor analysis and LUR methods to identify elemental and organic components spatially correlated with variation in diesel-related sources across a downtown area. To do so, we assessed a suite of elemental and organic constituents, including polycyclic aromatic hydrocarbons (PAHs), hopanes, and steranes, and implemented factor analyses including both elemental and organic constituents of PM_2.5_ separately for winter and summer, and merged across seasons. We hypothesized: (1) that we would observe distinct factors (spatially-correlated suites of constituents) with substantial loadings of constituents previously associated with diesel-related sources in the literature, and (2) that spatial variability in factors could be predicted using GIS-based source terms and LUR modeling methods.

## 2. Materials and Methods

### 2.1. Study Design

Monitoring and data collection methods are detailed in two prior manuscripts from this study [26,27]. Briefly, we aimed to: (1) capture spatial variability in various traffic-related sources (esp., diesel emissions), and (2) to use highly-resolved GIS-based indicators (e.g., total traffic density, total truck density, bus route frequency) to distinguish several types of diesel emissions from other urban sources. We did so by allocating 36 sampling sites across a small (2.8 km^2^) domain in downtown Pittsburgh, to capture source-driven variation in three GIS-based indicators of local pollution: vehicular traffic, truck traffic, and bus route frequency. These three source parameters were developed using GIS, based on Pennsylvania Department of Transportation (PennDOT) Annualized Average Daily Traffic counts and Google transit bus routes to derive three kernel-weighted density surfaces. Each category was dichotomized at the median, and cross-stratified to create eight sampling classes (e.g., high diesel/ high truck/low bus density, etc.), and a comparable number of sampling sites were randomly selected from each class, while ensuring spatial coverage across the downtown area.

At each site, during one randomly-selected week in each sampling season, programmable monitors were used to collect integrated samples of PM_2.5_ and constituents using Harvard Impactors (HIs) (Air Diagnostics and Engineering, Inc., Harrison, ME, USA) with 37-mm Teflon™ filters (PTFE membrane, 2-µm pores, Pall Life Sciences) housed in weather tight Pelican boxes.

In separate Pelican boxes, organic compounds were collected using cyclone-adapted HIs (Air Diagnostics and Engineering, Inc.) with pre-baked 37-mm quartz fiber filters (Pallflex Tissuquartz non-heat-treated filters, Pall Life Sciences). Prior to deployment, quartz fiber filters were placed into porcelain dishes using Teflon-coated tweezers and baked for four hours at 900 °C (Thermo Scientific Thermolyne oven, Waltham, MA, USA) to remove trace organics. During retrieval, the quartz filters were removed from the cyclone-adapted HI, enclosed in a petri dish, placed inside an insulated box with ice packs, and covered in foil to prevent light exposure. Quartz filters were stored in foil-wrapped petri dishes at −20 °C, until shipped overnight on ice for analysis.

All particle samplers (using either Teflon or quartz filters) were programmed using a chrontroller (ChronTrol Corporation, San Diego, CA, USA), which turned the monitor’s pump on for a selected subset of hours during the week (Monday through Friday, 7 a.m. to 7 p.m.), simultaneously across all 36 distributed monitoring locations. Battery-operated vacuum pumps (SKC, Inc., Eighty-Four, PA, USA) were calibrated to 4.0 L/min. Flow rates were temperature-adjusted based on weather forecasts prior to each deployment.

Winter sampling was performed from 14 January to 3 March, and summer sampling from 10 June to 2 August 2013. Four reference sites (one upwind site and three sites within the sampling domain) were monitored during every sampling session to temporally-adjust samples collected across different weeks. Each sample from the 36 distributed sites was temporally-adjusted by dividing the observed concentration by the mean concentration from the four reference sites during that week, then multiplying the result by the mean concentration from the four reference sites for the entire sampling season. We thereby estimated the expected seasonal average concentration at each distributed sampling site, enabling direct comparisons across sampling locations. These temporal adjustment methods are further detailed in Tunno et al. 2018 and Shmool et al. 2014 [26,28].

### 2.2. Tracer Selection

Based on our previously-published literature review of elemental source tracers [25], only 3 elemental constituents [aluminum (Al), calcium (Ca), and iron (Fe)] were identified in diesel emissions in two or more studies. However, to provide source contrast for factor analysis, we also examined elemental constituents associated with other urban emission sources relevant to spatial variation in pollution across our region (e.g., gasoline emissions, non-tailpipe vehicular emissions, industrial emissions related to steel-making and coal, fuel oil burning). The literature review identified the following tracers for vehicular tailpipe and non-tailpipe emissions: black carbon (BC), Ca, Fe, zinc (Zn) (motor vehicles); barium (Ba), copper (Cu), Fe, molybdenum (Mo), antimony (Sb), strontium (Sr), Zn (brake/ tire wear); Al, Ca, Fe (soil/ road dust resuspension); and BC, Al, Ca, Fe (diesel emissions). Constituents associated with long-range transport of pollution into our region included arsenic (As), nickel (Ni), selenium (Se); these species, along with sulfur (S), are commonly associated with coal combustion/coal-burning power plants. The Pittsburgh area, however, contains one of the country’s largest coke works, complicating the interpretation of this tracer in our study area. Fe, manganese (Mn), lead (Pb), and Zn have been associated with steel manufacturing. Nickel (Ni) and vanadium (V) have been associated with residual fuel oil/heating oil combustion.

To select organic markers, given the relatively greater instability of these compounds, we considered additional criteria, ensuring that each was: (1) previously identified, in the published literature, as a marker of diesel exhaust, (2) quantifiable using thermal desorption gas-chromatography mass-spectrometry (TD-GC-MS) or other GC-MS method, and (3) had relatively lower volatility and reactivity, in comparison to other diesel exhaust components (e.g., four or more aromatic rings, higher molecular weight) [29,30,31,32,33,34,35]. Our final list included nine PAHs (benz[a]anthracene, benzo[a]pyrene, benzo[e]pyrene, benzo[ghi]fluoranthene, benzo[ghi]perylene, chrysene, fluoranthene, indeno[123-cd]pyrene, and pyrene), hopanes (homohopane, hopane, norhopane, trisnorhopane), and steranes (cholestanes). A few more reactive compounds previously associated with diesel (i.e., pyrene, benz[a]anthracene, and benzo[ghi]perylene) were also retained for source apportionment.

Finally, several gases normally used as tracers for vehicular emissions were measured, as described in the next section—including nitrogen dioxide (NO_2_) and volatile organic compounds (VOCs) [i.e., benzene (BENZ), toluene (TOL)].

### 2.3. Sample Analysis

To determine PM_2.5_ concentrations, Teflon™ filters were pre- and post-weighed using an ultramicrobalance (Mettler Toledo Model XP2U, Columbus, OH, USA ) inside a temperature and relative humidity-controlled glove box (PlasLabs Model 890 THC, Lansing, MI, USA). Black carbon (BC) was estimated using an EEL43M Smokestain Reflectometer (Diffusion Systems Limited, London, England) [36] and reported in absorbance units (abs) [37].

For elemental composition, inductively-coupled plasma mass spectrometry (ICP-MS) analyses were conducted on all Teflon filters used to collect PM_2.5_, by the Wisconsin State Laboratory of Hygiene following documented protocols (ESS INO Method 400.4; U.S. EPA Method 1638) [38]. Using ICP-MS, every filter was analyzed for 25 elements: Al, As, Ba, Ca, Cd, Ce, Cr, Cu, Cs, Fe, K, La, Mg, Mn, Mo, Ni, P, Pb, S, Sb, Se, Sr, Tl, V and Zn.

For OC and EC, thermal-optical reflectance on all quartz filters was performed at Desert Research Institute (DRI, Reno, NV, USA) [39]. Total carbon was calculated as the sum of total EC and total OC. Thermal desorption gas-chromatography mass-spectrometry (TD-GC-MS) was conducted by DRI [40] for all selected organic compounds, including polycyclic aromatic hydrocarbons (PAHs) [benz[a]anthracene (BAA), benzo[a]pyrene (BAP), benzo[e]pyrene (BEP), benzo[ghi]fluoranthene (BGHIF), benzo[ghi]perylene (BGHIP), chrysene (CHRYS), fluoranthene (FLRT), indeno[123-cd]pyrene (IP), and pyrene (PYR)], hopanes (homohopane (HOP9_10), hopanes (HOP6_7_8), norhopane (HOP3_4_5), trisnorhopane (HOP1_2), and steranes (four cholestanes). We also created a “total PAHs” indicator by summing the nine PAHs, and similar “total hopanes” and “total steranes” indicators.

Gases which were monitored using Ogawa passive badges (i.e., NO_2_), were analyzed using water-based extraction and spectrophotometry [41] (Thermo Scientific Evolution 60S UV-Visible Spectrophotometer, Waltham, MA, USA) for nitrogen dioxide (NO_2_) concentrations (ppb). Shelters and Radiello passive diffusive badges (Eurofins Air Toxics, Inc.) (Folsom, CA, USA) were used to collect and determine benzene (BENZ) and toluene (TOL) concentrations (µg/m^3^) [42].

### 2.4. Source Apportionment

Following the methods previously outlined in Clougherty et al. 2009 and Tunno et al. 2015, we performed a two-stage analysis wherein we: (1) use unconstrained factor analysis to identify groupings of spatially-correlated constituents, and (2) derive factor scores for each location, which are then mapped across the study area and modeled using LUR, to identify key sources that explain variance in concentrations.

We first performed season-specific unconstrained factor analysis on temporally-adjusted elemental and organic concentrations, to derive factors representing latent emissions source groupings. Second, we performed a merged (two-season) factor analysis including all 72 samples from the 36 sites. Every factor analysis included data from all 36 monitoring locations, and all 48 pollutants (25 elemental constituents, nine PAHs, four specific hopanes, total hopanes, total steranes, total PAHs, total carbon, total EC, total OC, NO_2_, BC, benzene, toluene).

To select an optimal number of factors, we considered eigenvalue-one criterion and scree plots, and retained factors explaining at least 2% of total variance. Constituents loading greater than or equal to 0.60 were included on each factor. The factor solution was sensitivity-tested using constituent loading cut-points of 0.50 and 0.70 to identify changes in groups, and to interpret factors. Summary factor scores were calculated for each factor, for each distributed monitoring location, each season, as well as merged across seasons. We used the U.S. EPA’s Positive Matrix Factorization (PMF) model version 5.0 as a sensitivity-test, to corroborate the observed factor solution [43].

Using LUR methods and sensitivity analyses detailed in Clougherty et al. 2013 [44] and Tunno et al. 2015 [45], we modeled factor scores as a function of GIS-based local emissions source indicators (Table 1). Separate LUR models for each factor were implemented using manual forward step-wise linear regression, separately for each sampling season, then in a merged-seasons model. Final LUR models for each factor retained only source covariates significant at *p* < 0.05, which increased R^2^ by at least 0.02, and which produced a variance inflation factor (VIF) of less than 1.

Finally, elemental and organic constituents loading strongly onto each factor were compared to the literature review, to identify potential sources. Source covariates identified as significant in the LUR for each factor were compared to the literature-based assessment, to corroborate these factor interpretations.

Factor analyses were performed using PROC FACTOR, and LUR modeling was performed using PROC MIXED, in SAS v. 9.3 (Cary, NC, USA). R statistical software v.3.1.2 (R Foundation for Statistical Computing, Vienna, Austria) was used to produce factor loading plots.

## 3. Results

### 3.1. Factor Analysis/Source Apportionment

**Figure 1:**
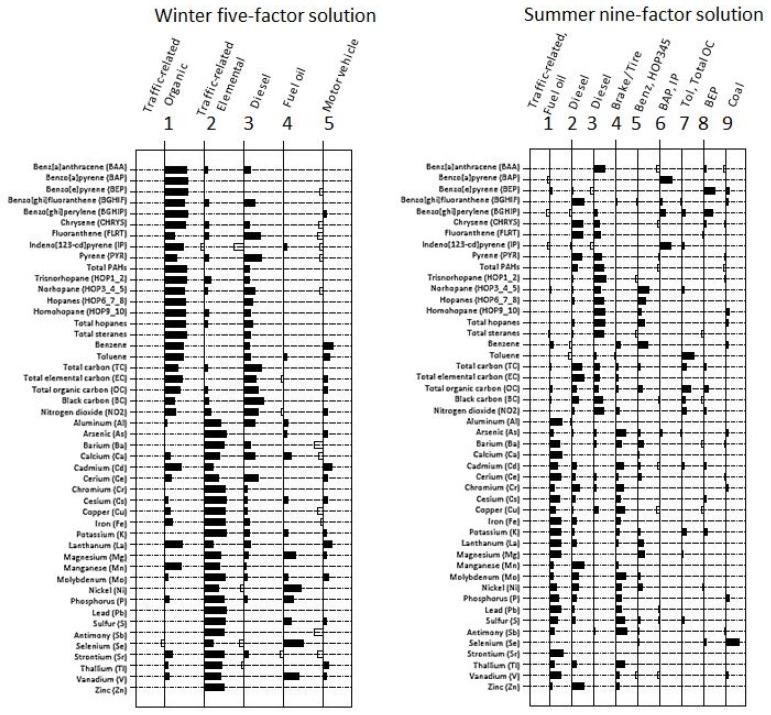
Factor loadings plot for winter five-factor and summer nine-factor solutions (*n* = 36).

We identified a five-factor solution for winter (*n* = 36) and a nine-factor solution for summer (*n* = 36) (Figure 1). Similar overall variance (%) was explained by seasonal factor solutions. In each season, we were able to identify diesel factors. For combined winter and summer, we identified an eight-factor solution, explaining 84% of overall variance.

For winter samples, a five-factor solution explained 88% of overall variability across constituent concentrations. Factor one (which explained 50% of total variance) was characterized by several organic compounds, and interpreted as ‘traffic-related organic.’ Factor two (24%), interpreted as ‘traffic-related elemental’, was characterized by elemental constituents (Figure 1). Factor three (7%) included ‘diesel tracers’ (BC, fluoranthene, NO_2_, pyrene, total carbon). Factor four (4%) included ‘fuel oil tracers’ (Ni, V), while factor five (3%) suggested ‘motor vehicle’ (benzene, Cd, La, toluene).

For summer samples, a nine-factor solution explained 88% of variability across all constituents (Figure 1). Factor one (38%) was interpreted as ‘traffic-related elemental’, as several elemental constituents loaded strongly, and also included ‘fuel oil tracers’ (Ni, V). Factor two (18%) suggested ‘diesel tracers’ (benzo[ghi]fluoranthene, chrysene, Cr, fluoranthene, Mn, pyrene, total carbon, total EC, Zn). Factor three (9%) also included ‘diesel’ (benz[a]anthracene, BC, hopanes, NO_2_, total PAHs, total steranes). Factor four (6%) was characterized by an indicator of ‘brake/tire wear’ (Cu, Mo, Sb). Factor five (4%) included benzene and norhopane, whereas factor six (3%) included benzo[a]pyrene and indeno[123-cd]pyrene. Factor seven (3%) included toluene and total OC. Factor eight (3%) included benzo[e]pyrene. Factor nine (2%) included coal-related tracers (i.e., Se).

**Figure 2:**
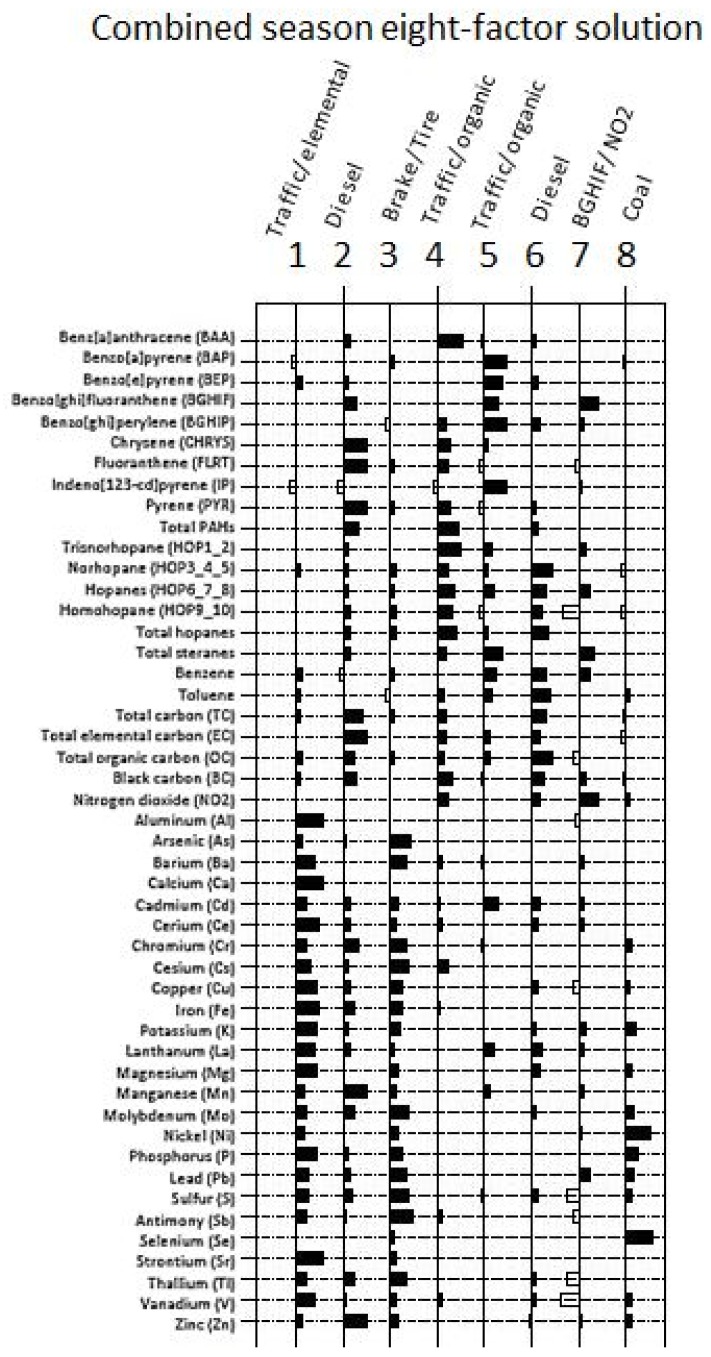
Factor loadings plot for eight-factor solution based on combined winter and summer pollutants (*n* = 72).

For combined samples (*n* = 72 samples: one from each of the 36 sites, over two seasons), factor one (36%) was interpreted as ‘traffic-related elemental’, as several elemental constituents loaded strongly (Figure 2). Factor two (16%) suggested ‘diesel’ (chrysene, fluoranthene, Mn, pyrene, total carbon, total EC, Zn). Factor three (11%) was characterized by tracers of ‘brake/tire wear’ (Cu, Mo, Sb). Factor four (7%) suggested ‘traffic-related organic’ (benz[a]anthracene, hopanes, total PAHs). Factor five (5%) contained ‘traffic-related organic’ compounds (benzo[a]pyrene, benzo[e]pyrene, benzo[ghi]perylene, indeno[123-cd]pyrene, total steranes). Factor six (4%) indicated ‘diesel’ (norhopane, toluene, total hopanes, total OC). Factor seven (3%) was characterized by benzo[ghi]fluoranthene and NO_2_. Factor eight (3%) included coal-related tracers (i.e., Se).

### 3.2. LUR Models for Factor Scores

**Figure 3:**
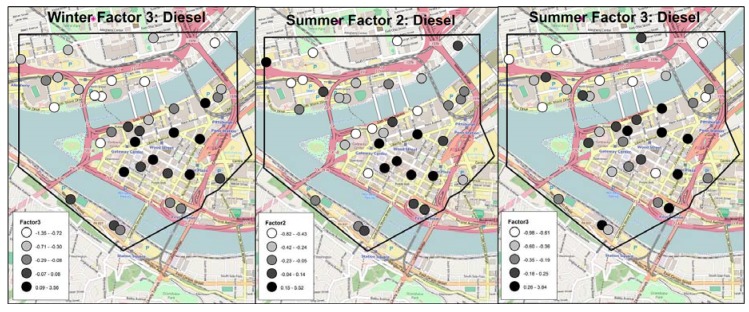
Spatial distribution of factor scores across monitoring locations for diesel-interpretsed factors (winter factor 3, summer factors 2 and 3).

For winter samples, variability in the ‘traffic-related organic’ factor was explained by building density within 75 m, and parking garages within 125 m (Table 2). Bus density within 50 m and truck density within 200 m explained variability in the ‘diesel’ factor. Higher factor scores were observed at sites within the downtown core, compared to those further away (Figure 3). Signaled intersections within 125 m and commercial land use within 50 m were significant for the ‘motor vehicle’ factor. Factor two, comprising ‘traffic-related elemental’ constituents, had no significant predictors, nor did the ‘fuel oil’ factor.

For summer samples, bus stop use within 100 m was significant in the final LUR for the ‘traffic-related elemental’ factor (Table 3). Bus density within 50 m was significant in the final model for the first ‘diesel’ factor; and truck density within 200 m for the second ‘diesel’ factor. For both diesel factors, higher factor scores were observed at sites within the downtown core, compared to those further away, as in winter (Figure 3). The ‘brake/tire wear’ factor was predicted by primary and secondary roadway length within 125 m. Truck density within 25 m and bus stop use within 100 m were significant for the ‘benzene/norhopane’ factor. Impervious area within 50 m and length of primary roads within 125 m explained significant variability in the ‘benzo[a]pyrene and indeno[123-cd]pyrene’ factor. Commercial land use within 200 m predicted the ‘benzo[e]pyrene’ factor. No significant predictors were identified for the ‘toluene/total OC’ and ‘coal’ factors.

For combined samples, the ‘traffic-related elemental’ factor was characterized by signaled intersections within 125 m (Table 4). The first ‘diesel’ factor was predicted by mean bus density within 200 m and commercial land use within 25 m. The ‘brake/tire wear’ factor was characterized by primary and secondary roadway length within 50 m, whereas the ‘traffic-related organic’ factor was predicted by traffic density within 200 m. Factor five, the other ‘traffic-related organic’ factor, was predicted by primary roadways within 125 m. Bus density within 200 m characterized the second ‘diesel’ factor. The ‘benzo[ghi]fluoranthene/NO_2_’ factor was predicted by temperature and bus density within 200 m. The ‘coal’ factor (Ni and Se) was predicted by commercial land use within 25 m, building density within 25 m, and railroad length within 200 m.

### 3.3. Sensitivity Analysis

Varying the factor loading cut-point to 0.50 or 0.70, the elimination or addition of constituents to any factor were minimal, and did not alter the factor interpretations based on the literature review. Using U.S. EPA’s PMF model, factor interpretations remained similar to those determined using the unconstrained factor analysis. Sensitivity testing of LUR models did not alter identification of key sources predicting variability in each factor.

## 4. Discussion

To identify suites of diesel-related constituents, and to identify key sources contributing to spatial variation in these constituents and overall PM_2.5_, we combined elemental and organic constituents in factor analyses for winter, summer, and merged across seasons. As hypothesized, we were able to successfully observe distinct factors related to diesel, and to use LUR modeling to identify key sources explaining spatial variation in these factors.

Distinct factors characterized by constituents previously associated with diesel emissions were observed in both the winter and summer seasons. In winter, one factor—consisting of BC, fluoranthene, NO_2_, pyrene, and total carbon—was interpreted as “bus- and truck-related,” and corroborated by LUR, in that both bus and truck densities explained significant spatial variance (R^2^ = 0.75). In summer, we identified two diesel-related factors: one bus-related (characterized by benzo[ghi]fluoranthene, chrysene, Cr, fluoranthene, Mn, pyrene, total carbon, total EC, and Zn), and one truck-related (characterized by benz[a]anthracene, BC, hopanes, NO_2_, total PAHs, and total steranes). As hypothesized, LUR models using GIS-based emissions source covariates roughly corroborated these factor interpretations; bus density explained significant variation in the bus-related factor, and truck density explained variation in the truck-related diesel marker. Merging across seasons, we identified an eight-factor solution including two apparent diesel factors: the first consisted of chrysene, fluoranthene, Mn, pyrene, total carbon, total EC, and Zn; the second included norhopane, toluene, total hopanes, and total OC. Both were correlated with bus density.

We previously used LUR modeling to assess spatial variation in individual pollutants including PM_2.5_, BC, EC, and OC across this area, and found that bus-related source indicators explained more spatial variance in these pollutants than did other source terms [27].

Due to the complexity of PM_2.5_, changes in diesel technology over time, and the spatial variability in the pattern of multiple emissions sources across an intra-metropolitan city, characterization of DPM is quite challenging, and there is a need to validate better tracers and to explore the utilization of factor analysis and related methods, to identify suites of constituents that may provide a diesel emissions “signature.” Here, we found that diesel factors varied somewhat in the relative loadings of different constituents each season—as expected, given the complex nature of diesel emissions and the substantial role of meteorology in modifying the chemical fate and transport of diesel emissions—although signatures were consistent enough to suggest source identifiability in both seasons.

DPM is estimated to be predominantly (~80%) carbon [19,20,21], although EC and OC are not specific to DPM. Jin et al. (2014) found that total carbon accounted for approximately 82% of DPM mass in heavy-duty diesel engines [46], while Noll et al. (2015) indicated a strong correlation between total carbon and EC [47]. Here, we examined a wide suite of potential tracers in combination, and would recommend doing so wherever possible. Nonetheless, we found that total carbon was strongly associated with diesel in both seasons, in both the factor analysis and LUR, while associations with BC, EC, or OC were less consistent. As such, our results do not contradict the Mine Safety and Health Administration (MSHA) guidance on the use of total carbon as a diesel exhaust marker [48]. We also found substantial loadings of PAHs, including fluoranthene and pyrene; these PAHs, derived from coal tar, were consistently identified in our diesel-related factors, regardless of season or analytic approach, and thus may prove useful markers of DPM across urban areas [49,50,51].

By examining a large number of different diesel-related tracers, we were able to distinguish among specific diesel sources (e.g., bus vs. truck), and to more clearly separate their impacts from those of other urban sources (e.g., non-tailpipe emissions, heating oil emissions). While most of our resultant factors were “traffic-related,” the type of vehicular emissions did differ substantially (e.g., bus vs. truck-related diesel, gasoline emissions, brake/tire wear). As such, the novelty of this study is the ability to separate specific “diesel” factors from a larger suite of pollutants within a dense urban core, including both tailpipe and non-tailpipe vehicular emissions, and other urban sources (e.g., heating fuel oil); we did so via a process which leveraged spatial correlations among pollutants, the existing literature to provide preliminary interpretations of source factors, and LUR modeling (which spatially linked source factors to GIS-based source terms) to corroborate factor interpretations. We found that the bulk of spatial variance in downtown-area emissions was indeed diesel-related emissions (specifically, from diesel buses), and demonstrated the utility of the method to identify modifiable targets for policy intervention and emissions reduction. By better quantifying these source-specific impacts and their spatial patterns through factor analysis and LUR modeling, we can better support the development of policies and interventions to directly reduce diesel-related exposures and improve health in urban areas.

## 5. Conclusions

In sum, we found substantial spatial variance in multiple elemental and organic constituents of PM_2.5_ previously associated with diesel emissions, even across a very small urban core (2.8 km^2^). Factor analytic source apportionment analyses, both stratified by and merged across seasons, identified multiple diesel-related constituent suites, variously explained in LUR models by GIS-based indicators of truck density, bus density, and other diesel sources. Our approach identified key constituents, including specific PAHs, which may prove useful tracers for DPM exposures in future urban source apportionment and epidemiological studies.

## Figures and Tables

**Table 1:** GIS (Geographic information system)-based source density indicators used for LUR (land use regression) modeling of factor scores.

Source Category for LUR Modeling	Covariates Examined (All buffers tested from 25 m to 200 m)	Data Source
Traffic density indicators	Mean density of annualized average traffic Mean density traffic (primary and secondary roads) Number of signaled intersections Annualized average traffic/Aspect ratio	Pennsylvania Spatial Data Access (PASDA, 2014) Southwestern Pennsylvania Commission (SPC, 2011)
Road-specific measures	Mean beta index of road complexity and connectivity Distance to nearest intersection Number of intersections Distance to nearest major road Summed length of primary roadways Summed length of primary and secondary roadways Width of roadways	TeleAtlas StreetMap (2014) PASDA (2014)
Truck, Bus, and Diesel	Mean density of bus traffic Distance to nearest bus route Distance to nearest bus stop Bus stop use (total number of trips) Mean density of heavy truck traffic on nearest primary roadway	Google Transit Feed (7/14) PASDA (2014)
Industrial emissions	Mean density of SO_2_ emissions Mean density of PM_2.5_ emissions Mean density of NOx emissions Mean density of VOC emissions	National Emissions Inventory (NEI, 2011)
Land use/Built environment	Total area of commercial parcels Total area of industrial parcels Total area of industrial and commercial parcels Distance to nearest park Summed area of parks Building counts Distance to nearest building Mean percentage of imperviousness	Allegheny County Office of Property Assessments (AC OPA, 2013) SPC (2011) Allegheny County Department of Public Works (DPW) National Land Cover Database (NLCD, 2011)
Transportation facilities	Distance to nearest active railroad Summed line length of active railroads Distance to nearest bus depot Summed area of parking lots and garages Distance to river centerline	SPC (2011) Google Transit (2014) AC OPA (2013) PASDA (National Hydrography Dataset, 2014)
**Potential modifying factors**		
Structural modifiers	Aspect ratio: building height/ roadway width Mean building heights	DPW
Topography	Average elevation Average slope Mean percentage of tree canopy	National Elevation Dataset (NED, 2013) NLCD (2011)
Meteorology	Temperature Relative humidity Frequency of inversions Wind direction Wind speed	Obtained from sampler Univ. of Wyoming, Dept. of Atm. Science (2013) National Oceanic and Atmospheric Administration (NOAA, 2013)

**Table 2:** Winter factor score LUR (land use regression) results (*n* = 36). Percentage of explained variance is listed under each factor, along with proposed sources, final LUR covariates (with final R^2^), and covariates most strongly correlated with factor scores (rho).

Factor (% variance)	Proposed Sources	Final LUR Model Covariates (R^2^)	Covariates Most Strongly Correlated with Factor Scores (r)
1 (50%)	Traffic-related (organic compounds, benzene and toluene, Cd, La, Mn, total EC and OC, total hopanes, PAHs, and steranes)	Building density, 75 m Parking garages, 125 m (R^2^ = 0.30)	Building density (r = 0.44) Roadway width, 75 m (r = 0.39) Commercial land use, 75 m (r = 0.35) Parking garages, 125 m (r = 0.30)
2 (24%)	Traffic-related (elemental constituents)	No spatial covariates with *p* < 0.05	Distance near intersection (r = −0.36)
3 (7%)	Diesel (BC, fluoranthene, NO_2_, pyrene, total carbon)	Bus density, 50 m Truck density, 200 m (R^2^ = 0.75)	Bus density, 50 m (r = 0.83) Bus stop use, 200 m (r = 0.80) Truck density, 200 m (r = 0.63) Signaled intersections, 125 m (r = 0.62)
4 (4%)	Fuel oil (Ni, V)	No spatial covariates with *p* < 0.05	Tree canopy, 75 m (r = −0.33) Imperviousness, 150 m (r = 0.30)
5 (3%)	Motor vehicle (benzene, Cd, La, toluene)	Signaled intersections, 125 m Commercial land use, 50 m Distance near intersection (R^2^ = 0.44)	Signaled intersections, 125 m (r = 0.42) Commercial land use, 50 m (r = 0.42) Distance near intersection (r = −0.42) Building density, 25 m (r = 0.42)

**Table 3:** Summer factor score LUR (land use regression) results (*n* = 36).

Factor (% variance)	Proposed Sources	Final LUR Modeling Covariates (R^2^)	Covariates Most Strongly Correlated with Factor Scores (r)
1 (38%)	Traffic-related elemental Fuel oil (Ni, V)	Bus stop use, 100 m (R^2^ = 0.38)	Bus stop use, 100 m (r = 0.61) Bus density, 100 m (r = 0.53) Signaled intersections, 125 m (r = 0.44) Commercial land use, 200 m (r = 0.39)
2 (18%)	Diesel (benzo[ghi]fluoranthene, chrysene, Cr, fluoranthene, Mn, pyrene, total carbon, total EC, Zn)	Bus density, 50 m (R^2^ = 0.54)	Bus density, 50 m (r = 0.74) Bus stop use, 75 m (r = 0.74) Commercial land use, 175 m (r = 0.47) Signaled intersections, 75 m (r = 0.45)
3 (9%)	Diesel (benz[a]anthracene, BC, hopanes, NO2, total PAHs, total steranes)	Truck density, 200 m Aspect ratio, 50 m (R^2^ = 0.39)	Aspect ratio, 50 m (r = 0.53) Truck density, 200 m (r = 0.50) Traffic density, 200 m (r = 0.49)
4 (6%)	Brake/ tire wear (Cu, Mo, Sb)	Primary and secondary roadways, 125 m (R^2^ = 0.13)	Bus density (r = 0.38) Primary and secondary roadways, 125 m (r = 0.37) Truck density, 25 m (r = 0.36)
5 (4%)	Benzene, norhopane	Truck density, 25 m Bus stop use, 100 m (R^2^ = 0.21)	Bus stop use, 100 m (r = 0.31) Truck density, 25 m (r = 0.30) Parking garages, 150 m (r = 0.28)
6 (3%)	Benzo[a]pyrene, indeno[123-cd] pyrene	Imperviousness, 50 m Primary roadways, 125 m (R^2^ = 0.30)	Imperviousness, 50 m (r = 0.43) Primary roadways, 125 m (r = 0.38) Parking garages, 200 m (r = 0.35)
7 (3%)	Toluene and total OC	No spatial covariates with p < 0.05	Railroads, 200 m (r = 0.32)
8 (3%)	Benzo[e]pyrene	Commercial land use, 200 m (R^2^ = 0.11)	Commercial land use, 100 m (r = 0.33)
9 (2%)	Coal (Se)	No spatial covariates with p < 0.05	No covariates with r > 0.15

**Table 4:** Combined winter and summer factor score LUR (land use regression) results (*n* = 72).

Factor (% variance)	Proposed Sources	Final LUR Modeling Covariates (R^2^)	Covariates Most Strongly Correlated with Factor Scores (r)
1 (36%)	Traffic-related elemental	Signaled intersections, 125 m (R^2^ = 0.07)	Bus stop use, 100 m (r = 0.34) Signaled intersections, 125 m (r = 0.27) Commercial land use, 25 m (r = 0.25) Bus density, 25 m (r = 0.25)
2 (16%)	Diesel (chrysene, fluoranthene, Mn, pyrene, total carbon, total EC, Zn)	Bus density, 200 m Commercial land use, 25 m (R^2^ = 0.37)	Signaled intersections, 200 m (r = 0.57) Bus stop use, 200 m (r = 0.56) Bus density, 200 m (r = 0.55) Truck density, 200 m (r = 0.51)
3 (11%)	Brake/ tire wear (Cu, Mo, Sb)	Primary and secondary roadways, 50 m (R^2^ = 0.10)	Primary and secondary roadways, 50 m (r = 0.23)
4 (7%)	Traffic-related organic (benz[a]anthracene, hopanes, total PAHs)	Traffic density, 200 m (R^2^ = 0.06)	Traffic density, 200 m (r = 0.25) Primary and secondary roadways, 200 m (r = 0.25)
5 (5%)	Traffic-related organic (benzo[a]pyrene, benzo[e]pyrene, benzo[ghi]perylene, indeno[123-cd]pyrene, and total steranes)	Primary roadways, 125 m PM_2.5_ emissions, (R^2^ = 0.19)	Primary roadways, 125 m (r = 0.33) Road complexity, 150 m (r = 0.30) PM_2.5_ emissions (r = 0.30)
6 (4%)	Diesel (norhopane, toluene, total hopanes, total OC)	Bus density, 200 m (R^2^ = 0.10)	Bus stop use, 175 m (r = 0.35) Bus density, 200 m (r = 0.32) Truck density, 200 m (r = 0.30)
7 (3%)	Benzo[ghi]fluoranthene, NO_2_	Temperature Bus density, 200 m (R^2^ = 0.73)	Temperature (r = −0.81) Wind speed (r = 0.56) Bus density, 200 m (r = 0.23)
8 (3%)	Coal (Ni, Se)	Commercial land use, 25 m Building density, 25 m Railroads, 200 m (R^2^ = 0.11)	Commercial land use, 25 m (r = 0.40) Building density, 50 m (r = 0.33) Railroads, 200 m (r = 0.29)
